# Long-term outcomes of a pilot CT screening for lung cancer

**DOI:** 10.3332/ecancer.2010.186

**Published:** 2010-05-13

**Authors:** G Veronesi, P Maisonneuve, L Spaggiari1, C Rampinelli, G Pelosi, L Preda, F Petrella, A Borri1, M Casiraghi, R Bertolotti, N Rotmensz, M Bellomi

**Affiliations:** 1 Department of Thoracic Surgery; 2 Division of Epidemiology and Biostatistics; 4 Department of Radiology; 5 Department of Pathology, European Institute of Oncology, 20141 Milan, Italy; 3 School of Medicine, University of Milan, 20122 Milan, Italy

## Abstract

**Background:**

Low-dose computed tomography (CT) screening can detect early stage lung cancer in high-risk populations. However, no data on repeated annual screening over more than 5 years are available, and the impact of screening on lung cancer mortality is controversial.

**Methods:**

We analysed outcomes in high-risk asymptomatic volunteers (smokers and former smokers, >50 years) enrolled in a pilot study over 1 year from June 2000, who received annual low-dose CT for 7 years. Cumulative lung cancer incidence and survival were represented by Kaplan–Meier curves. Standardized incidence and mortality ratios were used to estimate risks relative to the general Italian and US population.

**Results:**

Compliance was 86% at the end of the seventh year in 1035 recruited volunteers (71% men, mean age 58 years). Lung cancer was diagnosed in 54 (5.3%); radical surgery was possible in 48/54 (87%); 39/54 (72%) had stage I disease. Five-year survival was 63% overall, 89% for stage I cases. During 6308 person-years of observation, 47 participants had died versus 75 expected in the Italian general population standardised for age and sex. Fourteen lung cancer deaths were registered versus 27 expected in a standardised US smoker population.

**Conclusions:**

Seventy percent of screening-diagnosed patients had stage I disease, and the survival of screen-detected cancer patients was high. Lung cancer mortality was favourable compared to age- and sex-matched population of US smokers, suggesting that mortality can be lowered by screening, although larger trials with longer follow-up are necessary to confirm these findings.

## Introduction

Lung cancer, already a leading cause of cancer death worldwide, is characterized by increasing incidence among women in western countries and among both sexes in developing countries [1, 2]. The disease is usually rapidly fatal from time of diagnosis not only because it is biologically aggressive, but also because it is typically diagnosed at an advanced stage [3]. As the chances of cure depend strongly on the stage of cancer at diagnosis [4] and because smokers are at greatly increased risk of lung cancer [5], anticipation of diagnosis by screening the high-risk population constitutes a potentially important tool for reducing lung cancer mortality. The most promising screening modality is low-dose spiral CT of the chest [6–8], since, with an examination lasting for a few seconds, the technique can identify lung nodules of just a few millimetre in diameter, without contrast, with low radiation exposure and limited costs. However, no data on the results of repeated CT screening over more than 5 years are available. Furthermore, data on the impact of CT screening on mortality are few and controversial. The encouraging results of I-ELCAP − in which patients with screening-detected lung cancer had estimated lung cancer-specific survival of 80% at 10 years [9] − contrast with the absence of evidence for reduction in lung cancer deaths when screening outcomes in pilot studies were compared with those predicted by models [10]. Nevertheless, a model predicting outcome using data from participants in the Mayo [8] CT-screening study [11] indicated a 28% reduction in the lung cancer mortality at 6 years due to screening, although the reduction in all-cause mortality was only 2% at 15 years due to increased mortality from non-lung cancer causes associated with smoking. We report on long-term outcomes in a screened population that has undergone seven or more annual screening scans for lung cancer, focusing on the survival and stage distribution at diagnosis of those diagnosed with lung cancer, and the disease-specific mortality and mortality for competing risks in the entire screened population.

## Methods

We enrolled 1035 high-risk volunteers in a pilot-screening study over 1 year, starting in June 2000. By August 2007, those enrolled had received the baseline low-dose CT scan plus seven annual screening scans. The eighth scanning round has been completed but data are not yet available. Recruitment criteria were: age >50 years, and a smoking history of more than 20 pack-years, with former smokers giving up smoking not more than 10 years previously. Subjects were recruited from all over the country but most were from Milan area in Italy. A single-detector scanner was used up to 2004, since then 8-or 16-detector scanners have been used.

The CT-screening parameters and workup algorithm have been published elsewhere [12]. Briefly volunteers with a nodule >5 mm were initially recalled for high-resolution CT and those with lesions >7 mm received PET (or PET/CT). After multi-detector CT screening was introduced, high-resolution CT was no longer performed and suspicious nodules >5 mm were assessed by CT/PET or repeated low-dose CT at 3-month intervals. The study was approved by our Institute’s Ethics Committee. All participants were informed about the methods and aim of the study and signed a written informed consent form.

## Statistical methods

### Evaluation of lung cancer incidence and mortality in comparison with background

We calculated the number of person-years at risk from the date of first CT until the date of last contact or death. The expected numbers of lung cancer cases and of deaths for any cause or specific causes were calculated by multiplying the number of person-years accumulated in each stratum of age and sex by the corresponding age- and sex-specific lung cancer incidence [13] and mortality rates reported for the general population in Italy [14]. Standardized incidence (SIR) and mortality ratios (SMR) − ratios of observed to expected cases/deaths − were used to estimate relative risks. The expected number of deaths due to lung cancer was also estimated from published age- and sex-specific mortality rates observed in the smokers participating in the US Cancer Prevention Study II [15, 16].

### Cumulative lung cancer incidence and survival

Cumulative lung cancer incidence and survival were represented according to Kaplan–Meier, and the log-rank test was used to assess the significance of differences between groups. Subjects were right-censored if they were alive at the end of the study period or alive at some time but later lost to follow up. The analyses were performed with SAS version 8.2 (Cary, NC). All *p* values refer to two-sided tests.

## Results

The enrolled population of 1035 consisted of 739 (71%) men and 296 (29%) women, of mean age 58 years (range 50–76), 890 (86%) of whom were smokers and 145 (14%) former smokers. At the end of March 2008, 852 had presented for the seventh screening scan. Study compliance was 852/988 (86.2%) (1035 recruited minus 47 deaths = 988). One hundred and eight smokers at baseline (13%) stopped smoking after enrolment and 14 (2%) former smokers started smoking again during follow-up. Fifty four (42 men, 12 women) were diagnosed with lung cancer. Mean age at diagnosis was 62 years (range 50–76). One interval cancer occurred (diagnosed between repeated scans due to occurrence of symptoms).

Forty-five patients received radical surgery (44 lobectomies; 1 segmentectomy). Eight patients (four diagnosed by mediastinoscopy, two by fine needle aspiration and two by thoracoscopic biopsy) received non-surgical treatment. One patient was treated elsewhere and the type of treatment is unknown.

According to the new TNM classification system (seventh edition) [17], disease stage at diagnosis is shown in Figure 1. Thirty-eight patients (70%) had stage I NSCLC (35 stage IA and 3 stage I B). The stage distribution of the patients at diagnosis is shown in Table 1 which indicates that on average the high proportion of stage I diagnoses was maintained over 7 years.

Morphologies were adenocarcinoma in 33 (61%), squamous cell carcinoma in 7 (13%), small cell carcinoma in 7 (13%), carcinoid in 2 (4%), and not otherwise specified non-small cell lung cancer in 5 (9%). Overall 5-year survival was 63%; survival for stage I A and B disease was 89% (Figure 2).

Ten patients (15.6%) were operated on for benign disease with no postoperative morbidity or mortality. Mean size of lung cancers at preoperative CT scan were 19 mm for baseline cancers and 14 mm for cancers detected at subsequent screening rounds.

In the absence of data on lung cancer incidence and mortality rates in a comparable population of non-screened smokers, we decided to compare the number of cases from lung cancer in our series with that expected applying age- and sex-specific cancer incidence reported in the general Italian population. This will represent only an indirect quantification of the risk since the prevalence of smoking in Italy was about 31% in men and 22% in women during the study period with approximately 20% of men and 10% of women reporting smoking more than 15 cigarettes per day [18]. During 6308 person-years of observation, 54 volunteers were diagnosed with lung cancer compared to 14 expected using the cancer rates observed in the Italian general population (SIR 4.0; 95% confidence interval [CI] 2.745.63).

Fourteen lung cancer deaths were observed in our series compared to 10 expected in the Italian general population however this includes only about 20% smokers versus 100% in our study (SMR 1.43; 96% CI 0.642.74) (Table 2). Therefore, in the screened series we had a standardised disease-specific mortality risk of 1.4 versus a standardised incident risk of 4.0. This represents an indirect sign of the beneficial effect of screening.

Note that the Italian general population is at lower risk of lung cancer incidence due to lower smoking prevalence (only 20% compared to 100% in the study population).

While cancer mortality rates in smokers were not available in Italy, we attempted to assess the benefit of screening using mortality and survival rates extrapolated from US smokers in the Cancer Prevention Study II [15]. After standardising for age and sex, the observed lung cancer deaths in our screened Italian population were significantly lower than expected in the unscreened US smoker population (14 observed versus 27 expected with an SMR 0.51 95% CI 0.23–0.98) (Table 3).

## Discussion

Studies of chest X-ray and sputum cytology screening for lung cancer, conducted in the 1970s, showed no mortality benefit [19–21]. Mass screening for lung cancer was therefore abandoned and is not currently recommended. Interest in lung cancer screening has revived recently however, because of reports indicating that low-dose CT can detect smaller and earlier stage lung cancers than conventional X-ray [6, 7]. Large-scale randomised controlled trials [22, 23] are currently in progress to determine the efficacy of low-dose CT-screening but definitive results will not be available for several years.

The main controversies surrounding lung cancer screening are over-diagnosis (identification of nonfatal cancers) [24] and the lack of demonstration of mortality reduction [10]. In this study, most (72%) cancers were stage I, confirming the potential benefit of screening in terms of chance of cure even after the first couple of screening rounds. Not more than 16% of symptom-detected cancers in the US are stage I and II according to SEER statistics [3]. Over the seven years of repeated screening in our study, the rate of diagnosis of early stage disease was maintained (Table 1).

This experience is in contrast to that reported in brief pilot studies [10, 12, 24] and emphasizes the importance of continuing annual screening for more than 5 years. Our experience in fact undermines the hypothesis that over-diagnosis is a problem with lung cancer screening. If over-diagnosis were a major phenomenon, the proportion of stage I cancers would be expected to decrease after the first couple of annual screening rounds, due to depletion of supposed non-fatal cases. Other evidence also indicates that non-fatal cancers form only a small proportion of lung cancers: almost all patients diagnosed with stage I lung cancer by chest X-ray screening and not treated, died of the disease [25–27]. Furthermore pathological and molecular analyses indicate that the morphology and genetic characteristics of screening-detected cancers are closely similar to those of symptoms-detected cancers [28–30].

In this study, the high overall (63%) and stage I survival (89%) at five years are consistent with the highly encouraging results of the I-ELCAP study [9] but at odds with the analysis of Bach et al. [10] which was applied to three pilot studies on lung cancer screening including the one we performed. This modelling analysis found that CT screening increased the rate of detection of lung cancers but did not much reduce the risk of advanced lung cancer or of lung cancer deaths. As we pointed out in [31], the analysis had limitations including too short a follow-up time and the exclusion the data of the first year of screening when comparing deaths. In fact, one of the authors of [10] emphasized that the model used could have missed a difference in mortality rate of up to 30%. (Does CT screening reduce Lung Cancer Mortality? James R. Jett, MD Speaker, Controversies Around Cancer Screening, Special Session, 2008; personal communication ASCO Annual Meeting.)

In the absence of data on lung cancer incidence and mortality rates in a comparable population of non-screened smokers, we compared the number of cases and deaths from lung cancer in our series with that expected applying age- and sex-specific cancer incidence and mortality rates reported in the general Italian population. This will represent only an indirect quantification of the risk since the prevalence of smoking in Italy was about 31% in men and 22% in women during the study period with approximately 20% of men and 10% of women reporting smoking more than 15 cigarettes per day [18]. During 6308 person-years of observation, 54 volunteers were diagnosed with lung cancer compared to 14 expected using the cancer rates observed in the Italian general population (SIR 4.0; 95% confidence interval [CI] 2.74–5.63).

It is worth noting that lung cancer incidence in our screened population was four-fold higher than that of the general Italian population due to the higher exposure to smoking while the risk of death was only 1.4-fold higher. This difference is probably due to a beneficial effect of screening.

While cancer mortality rates in smokers were not available in Italy, we attempted to assess the benefit of screening using mortality and survival rates extrapolated from US smokers in the Cancer Prevention Study II [15].

The comparison of mortality rate observed in our screened volunteers with [13] that expected in a population of US citizens of similar age, sex distribution and smoking habits confirmed a benefit of about 31–61% reduction in lung cancer deaths (SMR 0.51 95% CI 0.23–0.98) (Table 3). Although this direct comparison can be misleading since other competing risk factors are likely to differ between the two populations, it provides a further indication that lung cancer mortality may be reduced by screening. Our results are also in line with two other recent publications [11, 32]. In McMahon et al. [11], a micro-simulation model estimated a reduction in lung cancer-specific mortality by 28% at 6 years in subjects who received five annual CT scans compared to observation. The meta-analysis of Chien et al. [32] conducted on six studies indicated that CT can advance the diagnosis of asymptomatic lung cancers by 2 years compared to observation and reduce lung cancer mortality by 23% at 5 years, again compared to observation.

Two limitations of this pilot study is the absence of a true control group of subjects and the lack of an evaluation of psychological impact of screening.

Points of strengths are the single centre recruitment with high homogeneity in diagnosis and treatment, the high compliance and long duration of the study.

To conclude, our long-term study has shown that a high proportion (70%) of the cancers diagnosed over seven annual screening rounds were at stage IA and IB, and that overall actuarial 5-year survival of patients with screening-detected cancers was high (63%). Lung cancer mortality was favourable when compared both to the general Italian population and to an age- and sex-matched population of US smokers, indicating that low-dose CT screening of high-risk subjects may reduce lung cancer mortality.

## Figures and Tables

**Table 1:**
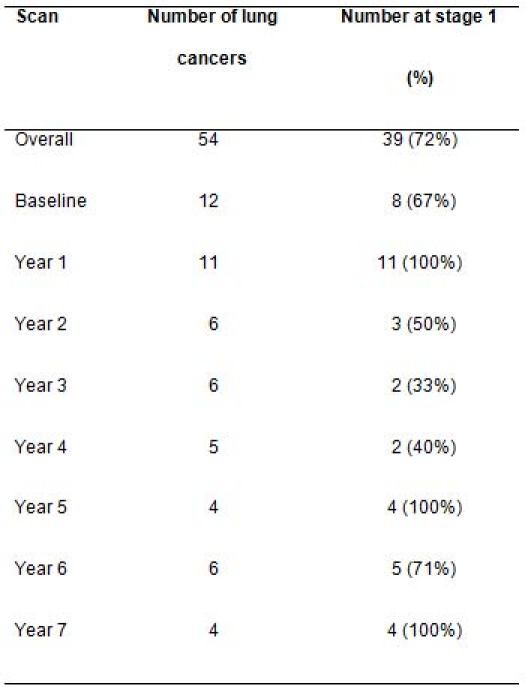
Numbers of lung cancers diagnosed, and number (with %) diagnosed at stage 1 over the period 2000 to 2008 in a high-risk population recruited to the CT-screening trial

**Table 2:**
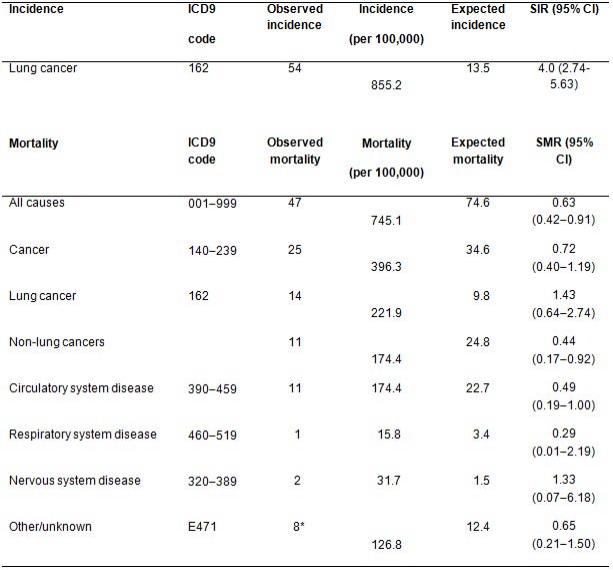
Observed lung cancer incidence and mortality during 6308 person-years of observation in a high-risk population recruited to CT-screening trial in comparison with expected incidence and mortality in the Italian general population (low risk)

ICD9: Ninth version of the International Classification of Diseases (WHO); SIR: standardized incidence rate; SMR: standardized mortality rates; CI: confidence intervals.

* Liver cirrhosis (*n* = 2), car accident (*n* = 2), foot gangrene (*n* = 1), renal insufficiency (*n* = 1), unknown (*n* = 2).

**Table 3:**
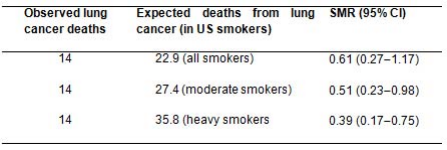
Observed lung cancer deaths during 6308 person-years of observation on a high-risk population recruited to CT-screening trial in comparison to expected lung cancer-specific death rate in a population of US smokers [18]

## References

[1] Jemal A, Siegel R, Ward E, Hao Y, Xu J, Murray T, Thun MJ (2008). Cancer statistics, 2008. CA Cancer J Clin.

[2] Enstrom JE, Heath CW (1999). Smoking cessation and mortality trends among 118,000 Californians, 1960–1997. Epidemiology.

[3] NCI: SEER Cancer Statistics Review

[4] Mountain CF (1997). Revisions in the International System for Staging Lung Cancer. Chest.

[5] Alberg AJ, Samet JM (2003). Epidemiology of lung cancer. Chest.

[6] Henschke CI, McCauley DI, Yankelevitz DF, Naidich DP, McGuinness G, Miettinen OS (1999). Early Lung Cancer Action Project: overall design and findings from baseline screening. Lancet.

[7] Sone S, Li F, Yang ZG, Takashima S, Maruyama Y, Hasegawa M, Wang JC (2000). Characteristics of small lung cancers invisible on conventional chest radiography and detected by population screening using spiral CT. Br J Radiol.

[8] Swensen SJ, Jett JR, Hartman TE, Midthun DE, Sloan JA, Sykes AM (2003). Lung Cancer screening with CT: Mayo Clinic Experience. Radiology.

[9] The International Early Lung Cancer Action Program Investigators (2006). Survival of patients with stage I lung cancer detected on CT screening. N Engl J Med.

[10] Bach PB Jett JR Pastorino U Tockman MS Swensen SJ Begg CB(200) Computed tomography screening and lung cancer outcomes JAMA 297 953 961 17341709 10.1001/jama.297.9.953

[11] McMahon PM, Kong CY, Johnson BE, Weinstein MC, Weeks JC, Kuntz KM (2008). Estimating Long-term Effectiveness of Lung Cancer Screening in the Mayo CT Screening Study. Radiology.

[12] Pastorino U, Bellomi M, Landoni C, De Fiori E, Arnaldi P, Picchio M (2003). Early lung-cancer detection with spiral CT and positron emission tomography in heavy smokers: 2-year results. Lancet.

[13] (2007). Cancer Incidence in Five Continents.

[14] ISTAT http://www.iss.it/site/mortalita.

[15] Thun M, Day-Lally C, Meyers D, Calle E, Flanders W, Namboodiri M, Burns D, Garfinkel L, Samet J (1997a). Monograph 8 In Changes in Cigarette-related Disease Risks and Their Implication for Prevention and Control.

[16] Thun MJ, Henley SJ, Calle EE (2002). Tobacco use and cancer: an epidemiologic perspective for geneticists. Oncogene.

[17] Detterbeck Fc, Boffa DJ, Tanoue LT (2009). The new lung cancer staging system. Chest.

[18] Gallus S, Colombo P, Scarpino V, Zuccaro P, Apolone G, La Vecchia C (2002). Smoking in Italy, 2002. Tumori.

[19] Marcus PM, Bergstralh EJ, Fagerstrom RM, Williams DE, Fontana R, Taylor WF (2000). Lung cancer mortality in the Mayo Lung Project: impact of extended follow-up. J Natl Cancer Inst.

[20] Kubik AK, Parkin DM, Zatloukal P (2000). Czech Study on Lung Cancer Screening: post-trial follow-up of lung cancer deaths up to year 15 since enrolment. Cancer.

[21] Marcus PM, Bergstralh EJ, Zweig MH, Harris A, Offord KP, Fontana RS (2006). Extended lung cancer incidence follow-up in the Mayo Lung Project and overdiagnosis. J Natl Cancer Inst.

[22] van Ierse CA, de Koning HJ, Draisma G, Mali WPTM, Scholten ET, Nackaerts K (2006). Risk-based selection from the general population in a screening trial: Selection criteria, recruitment and power for the Dutch-Belgian randomised lung cancer multislice CT screening trial (NELSON). Int J Cancer.

[23] Board of Scientific Advisors, National Cancer Institute (2005). Meeting Minutes.

[24] Jett JR (2005). Limitations of screening for lung cancer with low dose spiral computer tomography. Clin Cancer Res.

[25] Flehinger BJ, Kimmel M, Melamed MR (1992). The effect of surgical treatment on survival from early lung cancer. Implications for screening. Chest.

[26] Yankelevitz DF, Kostis WJ, Henschke CI, Heelan RT, Libby DM, Pasmantier MW (2003). Overdiagnosis in chest radiographic screening for lung carcinoma: Frequency. Cancer.

[27] Sobue T, Suzuki T, Matsuda M, Kuroishi T, Ikeda S, Naruke T (1992). Survival for clinical stage I lung cancer not surgically treated. Comparison between screen-detected and symptom-detected cases. The Japanese Lung Cancer Screening Research Group. Cancer.

[28] Pelosi G, Sonzogni A, Veronesi G, De Camilli E, Maisonneuve P, Spaggiari L (2008). Pathologic and molecular features of screening low-dose computed tomography (LDCT)-detected lung cancer: A baseline and 2-year repeat study. Lung Cancer.

[29] Belloni E, Veronesi G, Micucci C, Javan S, Scanagatta P, Taliento G (2008). Genomic characterization of early stage asymptomatic lung cancers. JCO.

[30] Bianchi F, Hu J, Pelosi G, Cirincione R, Ferguson M, Ratcliffe C, Di Fiore PP (2004). Lung cancers detected by screening with spiral computed tomography have a malignant phenotype when analyzed by cDNA microarray. Clin Cancer Res.

[31] Spaggiari L, Veronesi G, Bellomi M, Maisonneuve P (2007). Computed tomography screening for lung cancer. JAMA.

[32] Chien Chun-Ru, Hsiu-His Tony (2008). Mean sojourn time and effectiveness of mortality reduction for lung cancer screening with computed tomography. Int J Cancer.

